# Effect of Quaternary Ammonium-Based Antimicrobial Coating on the Mechanical Properties and Bacterial Adhesion to Gummetal Archwire

**DOI:** 10.1155/ijod/8157347

**Published:** 2025-03-11

**Authors:** Vincenzo Gramuglia, Steven Makowka, William Tanberg, Alan Zhou, Ashu Sharma, Thikriat Al-Jewair

**Affiliations:** ^1^School of Dental Medicine, State University of New York at Buffalo, Buffalo, New York, USA; ^2^Materials Testing Facility, School of Dental Medicine, State University of New York at Buffalo, Buffalo, New York, USA; ^3^Department of Biostatistics, School of Public Health and Health Professions, State University of New York at Buffalo, Buffalo, New York, USA; ^4^Private Practice, Brooklyn, New York, USA; ^5^Department of Oral Biology, School of Dental Medicine, State University of New York at Buffalo, Buffalo, New York, USA; ^6^Department of Orthodontics, School of Dental Medicine, State University of New York at Buffalo, Buffalo, New York, USA

**Keywords:** archwire, bacterial-adhesion, coating, elastic-modulus, friction, orthodontics, yield-strength

## Abstract

**Objectives:** This in vitro study investigated the mechanical and bacterial adhesion properties of Gummetal (GM) orthodontic archwire after application of quaternary ammonium compounds (QACs)–based antimicrobial coating.

**Methods:** Sixty orthodontic archwires were divided into three groups: coated GM (C-GM) group, consisting of a 0.016 × 0.022-inch GM core wire coated with QACs, and two control groups—one with a 0.016 × 0.022-inch uncoated GM wire and the other with a 0.016 × 0.022-inch stainless steel (SS) wire. The elastic modulus, yield strength, and static friction forces were compared between the C-GM and control groups. Measurements were conducted using a Dillon Quantrol TC2i universal testing machine. Surface roughness was evaluated using confocal profilometry, and bacterial adhesion was quantified through crystal violet dye staining.

**Results:** The mean elastic modulus for the C-GM, GM, and SS groups was 6.68 ± 0.1, 6.71 ± 0.2, and 19.7 ± 0.4 GPa, respectively, with significant differences observed between the C-GM vs. SS and GM vs. SS groups (*p*  < 0.001). The mean yield strength for the C-GM, GM, and SS groups was 7.5 ± 0.1, 7.6 ± 0.1, and 19.5 ± 0.2 N, with yield strength being significantly lower in the C-GM group compared to the GM (*p*=0.036) and SS (*p*  < 0.001) groups. For friction forces measured within metal brackets, the C-GM group exhibited a mean friction force of 1.0 ± 0.1 N, which was significantly lower than both the GM (1.1 ± 0.1 N, *p*=0.017) and SS (1.2 ± 0.1 N, *p*  < 0.001) groups. Confocal profilometry analysis indicated that the SS group had the lowest surface roughness, followed by GM and then C-GM. The mean bacterial count for the C-GM, GM, and SS groups was 0.605, 1.066, and 0.882 AU cm⁻^1^, respectively, with significant differences observed between each wire pair (adj. *p*  < 0.001).

**Conclusions:** The application of antimicrobial QACs to GM wires effectively reduced friction while preserving their strength and rigidity. Furthermore, the QAC coating demonstrated a significant reduction in bacterial adherence.

## 1. Introduction

The discovery of a new beta-titanium alloy, known as Gummetal (GM), is pivotal to advancing future orthodontic treatment. This wire has demonstrated numerous biomechanical properties that foreshadow its use in multiple phases of orthodontic treatment [1]. First, the composition of the wire includes titanium, niobium, tantalum, zirconium, and oxygen. These components provide strength, corrosion resistance, durability, and esthetics to GM [1]. Very importantly, this chemical makeup allows for high biocompatibility and effective nontoxicity of GM, especially with its lack of nickel and heavy metals [1]. To assist in expressing its properties, the wire is cold worked to develop the essential features applicable to treatment [2]. Some of these features include its remarkably elevated tensile strength and elasticity. Both features can be attributed to its exceptionally low Young's modulus [2]. Due to its low Young's modulus, GM exhibits greater elastic deformation under lower loads, meaning it has a higher tendency to stretch reversibly. This property enables GM to function effectively with a low load-to-deflection ratio throughout its use. Unlike stainless steel (SS) wire, GM does not undergo work hardening during application [2], allowing it to maintain its formability throughout treatment. This combination of properties is uncommon among orthodontic wires, making GM a uniquely beneficial material. Furthermore, GM exhibits high yield strain and superelastic deformation at room temperature [1], enabling the wire to endure significant stress and strain without failing.

The novel GM wire is also distinguished by its low frictional properties [3]. Its frictional output is comparable to both SS and NiTi wires, while significantly lower than that of TMA [3]. This characteristic suggests that GM wire could be highly effective across various phases of orthodontic treatment. Current investigations are exploring its use in the second and third stages of treatment. In stage two, molar relationship correction, space closure, and the adjustment of overjet and overbite are carried out [4]. In the third stage, finishing and detailing are performed [4].

GM wire is more elastic and ductile than SS and CoCr (Elgiloy), making it easier to bend and control [1]. Unlike SS, GM retains its elasticity throughout treatment, preventing stress-induced permanent deformation and enhancing its long-term functionality. This resistance to deformation makes GM particularly suitable for molar movement during space closure, as it is less prone to fracturing during stage two treatment. Additionally, GM's superelasticity allows the wire to return to its original shape after stress is applied, without energy loss, a property known as “without hysteresis” [1]. These attributes enable GM to be used effectively for longer durations, reducing the need for frequent wire changes.

Orthodontic treatment has been associated with the development of white spot lesions and the demineralization of tooth structure surrounding brackets [5]. Much of this is attributed to the microbial colonization of the orthodontic appliances. In response, researchers have begun exploring antimicrobial coatings on orthodontic archwires as a potential solution to inhibit bacterial colonization [6]. In one study, silver and zinc oxide coatings provided a robust antimicrobial effect on two of the most prevalent pathogenic bacteria associated with demineralization, *Streptococcus mutans*, and *Lactobacillus acidophilus* [6].

Quaternary ammonium compounds (QACs) have been utilized as antibacterial agents in diverse environments [7]. While numerous variants exist, a shared trait among many QACs is their cationic characteristics, as well as modified surfaces of long-alkyl chains to attract the negatively charged membranes of surrounding bacteria [7]. The positively charged QAC adheres to the anionic microbial cell membranes and induces lysis of the outer cell membrane, resulting in a bactericidal effect known as “Contact Killing” [7]. This mechanism would hinder the attachment of microbes to surfaces coated with QACs. Consequently, it would enable precise and targeted disruption of bacterial membranes with minimal impact on surrounding cells [7]. This is a very important implication for the potential use of QACs as a coating on medical and dental products.

One limitation of using an antibacterial coating is its potential impact on the wire's mechanical properties. Albawardi et al. [3] investigated the frictional forces generated by rhodium-plated GM wire, which is designed for esthetic purposes. The study revealed that the coated GM (C-GM) wire produced higher frictional forces compared to both SS and non-C-GM wires.

Zhou et al. [8] in a subsequent study investigated the mechanical, physical, and esthetic properties of GM archwires after the application of epoxy, polytetrafluoroethylene (PTFE), clear and white ceramic, and silicone in comparison to non-C-GM and SS. They found that the coatings improved some aspects of archwire properties but not all. Neither of the two studies assessed the antibacterial properties of the wires.

Microbial colonization on orthodontic appliances can lead to detrimental effects on tooth structure. Antibacterial coatings, while offering a promising solution by inhibiting bacterial growth, may pose the risk of compromising the mechanical integrity of the appliances. Moreover, the efficacy of an agent may be compromised after coating. To date, no studies have evaluated the impact of QAC coating on both bacterial colonization and the mechanical properties of GM wires. The aim of this in vitro study was to investigate the mechanical properties, as well as the ability to resist bacterial adhesion of GM wire after the application of QACs-based antimicrobial coating. It is hypothesized that there is no difference between the elastic modulus, yield strength, and frictional force of QAC-C-GM wires and control wires. Additionally, it is hypothesized that there is no difference in bacterial adhesion between the wire groups. The study was undertaken to determine if QAC coating would undermine the mechanical properties of the wires in any way, and additionally provide a significant resistance to bacterial growth and adhesion.

## 2. Materials and Methods

### 2.1. Framework

This in vitro comparative study utilized 60 orthodontic archwires divided into three groups (20 wires per group). An experimental group that included a 0.016 × 0.022-inch GM (GUMMETAL; JM Ortho corporation Lot # 200109M) wire coated with antimicrobial agent (C-GM) and two uncoated control wires composed of 0.016 × 0.022-inch uncoated GM and 0.016 × 0.022-inch SS (Ormco Co, Glendora, CA, Lot# 219-1408). Three-point bending, static friction, and bacterial count in association with the experimental wires were evaluated. Additionally, confocal profilometry was conducted to evaluate the surface characteristics of the three wire groups.

### 2.2. Sample Size Estimation

The sample size estimation was based on one-way ANOVA simulations to detect an approximate Cohen's *f* effect size of 0.40 and achieve a power of 80% with an alpha of 5%. The results were 20 samples in each of the three groups for a total of 60.

### 2.3. Coating Materials

The C-GM wire group was coated with covalently bonded QACs. Wires were coated by applied medical coatings (AMCs, Lockport, NY, USA). A precoating cleansing step of the GM wire was performed before the application of the antibacterial coating. It involved 7 min of ultrasonic cleaning, followed by further washing with deionized water and a generic cleaning solution. The GM wire was then air-dried followed by plasma treatment with an oxygen and hydrogen mixture for 5 min. The QAC antimicrobial overlay was then applied via spray and cured to the GM wire via heat catalysis to permit appropriate covalent bonding and allow for a uniform polymeric coating of the antibacterial substance on the GM wire.

### 2.4. Three-Point Bending Test

Following cleansing and application of the QAC antimicrobial coating, both the experimental and control wire groups underwent Three-point bend testing to compare their elastic modulus and yield strength. The test was carried out using a universal testing machine (Dillon Quantrol TC2i, Mecmesin, Dillon, Fairmont, MN) with a 3D-printed indenter and fulcrum (Formlabs Grey Pro Resin 3D-print via the Formlabs Form 3 printer) (Formlabs, Somerville, MA). SS metal contacts were used for the tips of the indenter and fulcrum (radii of the indenter and fulcrum were measured as 0.1 ± 0.05 mm). The test was performed utilizing all wires from each group, and a single new wire at every attempt. The room temperature was held at 25°C to prevent environmental bias. The wires were tested in 10 mm segments and were bent at a rate of 1.25 mm/min until they reached a “deflected distance” of 3.1 mm. Once the deflected distance was reached, bending forces were terminated and the wires returned to their original positions, as per BS ISO 15841-2014.

The relationship between the forces and bending distances of each test was evaluated by the formation of a graph with its *x*-axis being the distance each wire was bent (mm) and the *y*-axis being the total forces applied (Newtons). The graph was formed using the Emperor Test Software (Mecmesin, West Sussex, UK). The data from the graph were used to formulate the force deflection rate; information that would help calculate the elastic modulus of each group. To calculate the force deflection rate, the slope was measured from 0.25 to 0.75 mm. The yield strength of each group was also deduced from the same graph. The *y*-intercept of a parallel line which was placed at 0.1 mm offset from its original position was calculated to determine the yield strength. From these data, each group's stiffness relationship and strength were quantified. The experimental technique was conducted by one investigator (Alan Zhou).

### 2.5. Friction Test

The friction test was performed using the same universal testing machine mentioned previously. 0.022-inch slot maxillary right canine twin brackets with McLaughlin, Bennett, and Trevisi (MBT) zero-degree torque prescription were used. Two bracket materials were tested, metal (Victory Series, 3M Unitek, Monrovia, CA, LOT#MG5YD) and ceramic (Clarity Series, 3M Unitek, Monrovia, CA, LOT#MJ1AE). Two 3” × 1.5” x 1/8” precision ground aluminum plates (McMaster-Carr. Aurora, OH) were used as a foundation for the brackets during the friction test. The brackets were oriented and then bonded to the aluminum bases. The bonding was performed via coating the bases of all brackets with Henkel LOCTITE SF 713TM adhesive accelerator and Henkel LOCTITE superglue gel control. The brackets were oriented by strapping the aluminum plates to a 3D printed mounting jig made from Formlabs Form 3 printer (Formlabs, Somerville, MA) with 0-degree slits. Prior to adhesive placement, a straight 0.021 × 0.025-inch SS wire was engaged into the brackets. Each aluminum plate was set-up with four brackets for testing. A new SS wire was used after every five brackets. A custom setup of the universal testing machine was established to perform the friction test.

To evaluate the friction values of the wire groups, the straightest distal portion of each archwire was utilized. A three-prong plier was used to bend a hook into a single end of each wire being tested. After bending, each wire was ligated onto the brackets with clear elastic O-rings (3M AlastiK Easy-To-Tie Ligatures, Solventum, St. Paul, MN). A total of 150 g (100 g weight combined with a 50 g weight) of weights were added onto the hook. These weights were added to mimic the forces utilized in the clinical retraction of a maxillary canine. As the wire and bracket interacted, they were coated with artificial saliva (Pickering Laboratories, Mountain View, CA. LOT#2201043) three separate times with a small brush to replicate intraoral conditions. Wire pulling to instigate friction was performed at a 10 mm/min rate and evaluated utilizing the Emperor Force Software (Mecmesin, West Sussex, UK). The wires were pull-tested a total of three times, then another wire was loaded for the next section of testing.

### 2.6. Confocal Profilometry (Surface Roughness Measurement)

Confocal profilometry was performed using an Andor Dragonfly spinning disc confocal microscope, with a 40x/1.3NA oil objective, a 488 nm laser, and a *z*-step size of 160 nm. Three representative wires (one from each group) were chosen at random, then cut into 20 mm segments and attached to a glass slide using wax at each end of the wire. Wires were spaced ~15 mm apart from each other and then cleaned with isopropyl alcohol, followed by the application of oil for the immersion objective of the confocal microscope. Surface roughness analysis of the collected data was performed using the Fiji plugin TopoJ [9]. A roughness filter range from 2.5 to 250 μm was used according to ASME standard B46.1-2002 [10].

### 2.7. Bacterial Adhesion

The antibacterial coating on the C-GM wires was comprised of covalently bonded QACs which attract surrounding bacteria, fixes onto them, and causes the lysis of their outer cell membrane allowing for a bactericidal effect. The presence of the QACs was analyzed with the bromophenol blue (BPB) dye. Initially upon application, BPB appears purple but shifts to a blue color in the presence of QACs [11]. BPB appeared blue when in direct contact with the C-GM but retained its purple color upon contact with GM alone, confirming the presence of QACs on the C-GM wire.

To evaluate bacterial growth and adhesion on the wires, the wires in each group were cut into 25 mm segments and horseshoe bent at their midpoint (12.5 mm mark) using bird beak pliers. The groups were separated into three 10 mL glass test tubes and autoclave sterilized for 30 min at 121°C and 115 kPa.

Artificial saliva (Pickering Laboratories, Mountain View, CA) was placed into 50 mL conical centrifuge tubes and centrifuged for 20 min (10,000× *g* at 22°C) using a Sorvall Legend RT + centrifuge. Under a ventilated sterile culture hood, salivary supernatants were passed through 0.45-µm filters into a fresh 50 mL sterile tube. The wires were distributed into six 1.5 mL microfuge tubes containing 1 mL of filtered Saliva in each tube. The tubes were placed on a Thermolyne Labquake Shaker (Thermo Fisher Scientific, Waltham MA) and rotated for 30 min. After 30 min, the wires were removed and placed on sterilized glass microscope slides. The wires were allowed to air dry on slides for 30 min, establishing an even salivary coating.

**Figure 1:**
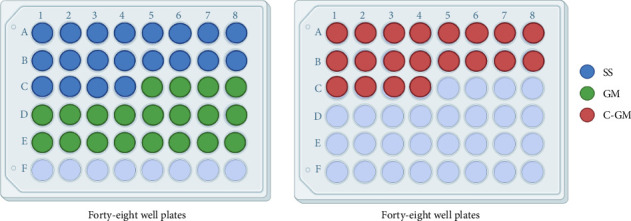
Forty-eight well plates layout with stainless steel (SS), gummetal (GM), and coated GM (C-GM) orthodontic archwires.

Under a ventilated culture hood, the wires were sorted into two 48 well plates (Figure 1). An aliquot of 0.75 mL of 1:10 diluted *S. mutans* late log phase cells in brain heart infusion (BHI) broth (Remel, Thermo Fisher) containing 1% sucrose was added to wells occupied by wires. The plates were placed into a sealed container with a Gaspak EZ Anaerobic Sachet to establish anaerobic conditions. The container was added onto a VWR Model 200 (VWR International, Radnor, PA) rocking platform in an incubator set to 37°C. The rocking platform was set to a gentle rocking motion as it tilted back and forth, mimicking a semidynamic environment.

After 5 days, the 48 well plates were removed from the incubator and the solution was removed from each well and disposed of leaving the orthodontics archwires with adhered bacterial cells (biofilm). The orthodontic archwires were placed into new 48 well plates in the same order. The wells with the wires were flushed twice with 1 mL of phosphate-buffered saline (PBS); each time shaking the wells briefly and aspirating off the unbound cells. Then, each well was filled with 0.75 mL of 0.1% crystal violet stain and incubated for 10 min at room temperature. After staining, the wells were flushed twice with 1 mL of PBS following the same procedure as described above. In total, 0.75 mL of acidic solution (30% acetic acid with 70% methanol) was then added to each well and agitated for 10 min at room temperature to extract stain bound to bacteria (biofilm). The bacterial adherence (biofilm) was quantified by measuring optical density at 540 nm using a Bio-Rad Smartspec 3000 spectrophotometer (Bio-Rad Laboratories, Hercules, CA).

### 2.8. Statistical Analysis

All data were tested for normality by either Shapiro–Wilks tests or by *Q*–*Q* plot in the case of the linear model. The elastic modulus and yield strength data failed to reject the null of normality in at least one of the wires. These two variables were then tested nonparametrically with pairwise Mann–Whitney tests and those *p*-values were adjusted for multiple testing by the Bonferroni method. The friction tests were evaluated with ANOVA and followed by post-hoc pairwise *t*-test and Bonferroni adjustment. The optical density data were first modeled with linear regression. The estimated marginal means contrasts from this model were evaluated by pairwise *t*-test with Bonferroni adjusted *p*-values.

## 3. Results

### 3.1. Elastic Modulus

Table 1 represents the elastic modulus of the three tested wire groups. The mean elastic modulus in the C-GM, GM, and SS groups was 6.6 ± 0.1, 6.7 ± 0.2, and 19.7 ± 0.4 GPa, with C-GM exhibiting the lowest average elastic modulus. The finding was significantly different in the C-GM vs. SS and GM vs. SS groups (*p*  < 0.001), but not between the C-GM vs. GM group.

### 3.2. Yield Strength

Table 2 represents the yield strengths of the three tested wire groups. The mean yield strength in the C-GM, GM, and SS groups was 7.5 ± 0.1, 7.6 ± 0.1, and 19.5 ± 0.2 N, with C-GM exhibiting the lowest yield strength. The finding was statistically significantly different in the C-GM vs. SS and GM vs. SS groups (*p*  < 0.001). There was also a statistically significant difference between the C-GM vs. GM group (*p*=0.036).

### 3.3. Friction Tests

The mean friction force in the metal bracket C-GM, GM, and SS groups was 1.0 ± 0.1, 1.1 ± 0.1, and 1.2 ± 0.1 N, respectively (Table 3). The C-GM group exhibited significantly lower friction forces than the GM (*p*=0.017) and SS (*p*  < 0.001) groups (Table 4). The mean friction force in the ceramic bracket C-GM, GM, and SS wire groups was 0.9 ± 0.1, 0.9 ± 0.1, and 0.9 ± 0.1 N, respectively. The ceramic brackets displayed nonsignificant results (*p*  > 0.05), leading to a failure to reject the null hypothesis.

### 3.4. Confocal Profilometry

**Figure 2:**
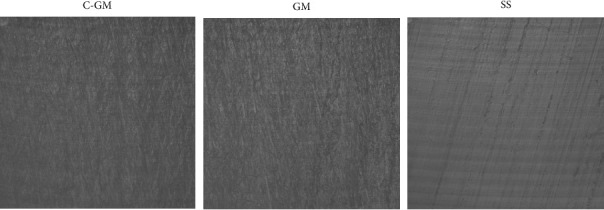
Confocal profilometry images.

The confocal profilometry root mean square (sq) in the C-GM, GM, and SS groups was 1.204, 0.509, and 0.254 µm (Table 5). This indicated that SS had the lowest surface roughness, followed by GM and C-GM.  Figure 2 depicts a 40x/1.3NA zoomed-in visual of the surface of each wire group. The surface of SS displayed less texture when compared to both C-GM and GM.

### 3.5. Bacterial Adhesion

**Figure 3:**
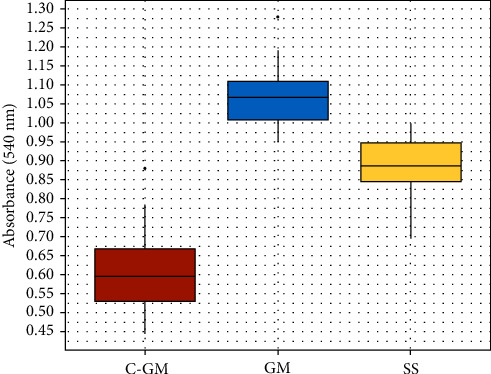
Box and whisker plots representing the absorbance values for bacterial adhesion according to wire type.

The mean bacterial count in the C-GM, GM, and SS groups was 0.605, 1.066, and 0.882 AU cm^−1^, respectively (ANOVA, *p*  < 0.001). Differences were also significant between each wire pair (adj. *p*  < 0.001) (Table 6 and Figure 3).

## 4. Discussion

This study evaluated the mechanical properties and bacterial count of GM wires coated with QACs. Given the findings of this study, the hypothesis was partially rejected, as the QAC-C-GM wire did not show a significant difference in elastic modulus compared to uncoated GM and SS wires. This is a crucial result, indicating that the coating does not compromise the wire's ability to deflect forces or resist deformation under stress. This finding reinforces the wire's maintained functionality, which is essential during the active phases of orthodontic treatment. Particularly, in cases requiring multiple bends and precise application for teeth alignment, the C-GM wire can deliver comparable results to an uncoated GM wire. This finding contradicts a previous study that used a functional bioactive glass (BG) coating [12]. In that study, BG coatings placed onto SS wires increased the elastic modulus of the wire, therefore altering the functional properties. The difference in findings between studies could be explained by the distinct materials utilized in each study. With this, the BG was coated onto its respective wires using electrophoretic deposition [12]. This technique differs from the curing technique used to apply QACs to wires in the current study.

This study found a statistically significant difference in the yield strength between the C-GM and the control wires, rejecting the initial hypothesis. This suggests that the C-GM wire cannot tolerate as much force as GM and SS wires. The implication of this is that if a C-GM wire was to be utilized in a more complex case with a higher force requirement, the difference in yield strength may become relevant. The findings in this study coincide with a previous study exploring functional coating onto core GM archwires [8]. The previous study showed statistically significant differences in the yield strength of both experimental and control GM wire groups after the application of PTFE, clear ceramic, white ceramic, and silicone coatings [8]. Future studies should review the maximum amount of force applied in complex orthodontic cases. Thus, the C-GM and GM wires should be tested and compared at the correlating force values. This will provide a better understanding of the C-GM wire's reliability and whether it is practical for specific treatment plans.

When tested on ceramic brackets, there was no statistically significant difference between the observed frictional forces of the C-GM and control wire groups, supporting the initial hypothesis. This raises the question of whether ceramic brackets contribute a similar frictional output on other orthodontic wire materials. However, when tested on metal brackets, C-GM significantly decreased friction compared to the noncoated controls. With C-GM displaying the lowest frictional output, these wires could potentially contribute to more efficient care during treatment.

The findings of this study align with previous research that demonstrated reduced frictional output in SS and titanium alloy orthodontic archwires when coated with materials, such as silver, zinc oxide, and aluminum oxide [12]. The applied QACs may belong to a specific group of lubricating functional coatings when used on orthodontic archwires. Further testing is necessary to determine whether similar friction-reducing effects are observed when antimicrobial QAC coatings are applied to other orthodontic wire materials, such as NiTi and SS.

Confocal microscopy revealed that the surface roughness of the C-GM wire exceeded that of the control wires. Previous studies suggested that surface roughness might be lower in coated wires, correlating with reduced frictional forces [12]. This unexpected increase in roughness raises important questions about the clinical efficacy of QAC-C-GM wires. For instance, a 2014 study found that plating an SS archwire with gold decreased overall surface roughness [12]. Although a different material was used, those results anticipated a different outcome than what was observed in this study. This discrepancy invites further exploration of whether increased surface roughness contributes similar effects across different orthodontic archwires. Future research should address these questions to advance the understanding of this topic. Additionally, the technique of QAC application may have contributed to the observed roughness and uneven coating of the C-GM wire. Investigating alternative application methods and assessing the consistency of surface roughness along the entire wire would be beneficial.

The significant differences between the bacterial count on each wire group do not support the second hypothesis. These findings are consistent with multiple studies in which bacterial count reduction was observed after applying a functional coating to orthodontic appliances. For example, in one study, significantly decreased amounts of *S. mutans* were seen in association with an Invisalign aligner coated with applied quaternary ammonium (QA)-modified gold nanoclusters [13]. A second study demonstrated reduced bacterial adherence when comparing epoxy resin-coated experimental NiTi wires with noncoated controls [14]. Similarly, another study found that applying Titanium Dioxide to SS wires decreased the adhesion of *S. mutans* [15]. Furthermore, research on nanocomposite coatings applied to Ti6Al4V/TiO2 substrates revealed a substantial reduction in *E. coli* bacteria after coating [16].

The finding that C-GM wires exhibited the lowest count of quantifiable bacteria is significant. Reduced bacterial attachment on the wire could translate to a lower risk of caries development on adjacent teeth and diminished wire build-up, potentially leading to fewer complications during orthodontic treatment. However, with these intriguing findings, it must be noted that the results associated with the C-GM wire displayed the most variability in quantifiable bacteria attached, whereas the SS wires displayed the most consistent amounts. The reasons for this increased variability are unknown. It is possible microbial interactions with QACs and/or differences in QAC deposition on the wire might have contributed to the variability.

The promising results of this study open several critical avenues for future research. A primary focus should be the longevity of antibacterial coating effectiveness in the dynamic oral environment, especially after repeated exposure to toothbrushing and other oral hygiene practices. Assessing these factors is essential for determining the long-term biocompatibility and durability of QAC-coated orthodontic wires. Moreover, future studies should explore the viability of *S. mutans*, as this study quantified bacterial presence using absorbance readings but did not confirm whether the bacteria were still viable. Understanding bacterial vitality will provide deeper insights into the true efficacy of the antibacterial coating in clinical settings.

Future research should also explore the application of QAC coatings to SS and NiTi wires to determine whether similar friction reducing and antibacterial effects can be achieved as those observed with GM wires. Another aspect that warrants examination is the thickness of bacterial biofilms, which was not assessed in this study. Biofilm thickness plays a significant role in the biofilm ecosystem function and community composition [17]. Future studies should evaluate biofilm thickness and its correlation with the mechanical performance of orthodontic wires.

## 5. Conclusions

Coating GM wires with antimicrobial QACs decreased friction and allowed them to maintain much of their strength and rigidity. The results also showed evidence supporting the effectiveness of the QAC coating in decreasing bacterial adherence.

## Figures and Tables

**Table 1:** Elastic modulus (GPa) of coated GM, GM, and SS.

Wire type	Mean	SD	Median	IQR	Min	Max	*p*-Value*⁣*^*∗*^		
							C-GM vs. GM	C-GM vs. SS	GM vs. SS
C-GM	6.68	0.06	6.71	0.09	6.57	6.75	1	<0.001	<0.001
GM	6.71	0.2	6.71	0.24	6.01	6.93	—	—	—
SS	19.68	0.31	19.66	0.43	19.21	20.22	—	—	—

*⁣* ^*∗*^Mann–Whitney test; *p*-value set at 0.05.

**Table 2:** Yield strength (N) of coated GM, GM, and SS.

Wire type	Mean	SD	Median	IQR	Min	Max	*p*-Value*⁣*^*∗*^		
							C-GM vs. GM	C-GM vs. SS	GM vs. SS
C-GM	7.5	0.07	7.53	0.06	7.38	7.66	0.036	<0.001	<0.001
GM	7.6	0.14	7.54	0.15	7.43	8.01	—	—	—
SS	19.25	0.2	19.22	0.32	18.91	19.63	—	—	—

*⁣* ^*∗*^Mann–Whitney test; *p*-value set at 0.05.

**Table 3:** Friction test (N) results of ceramic and metal brackets on coated GM, GM, and SS.

Bracket type	Wire type	Mean	SD	Median	IQR	Min	Max	ANOVA		
								*df*	*F*	*p*-Value*⁣*^*∗*^
Ceramic brackets	C-GM	0.85	0.09	0.84	0.09	0.7	1.01	2	2.397	0.110
	GM	0.89	0.06	0.87	0.06	0.79	1.01	—	—	—
	SS	0.94	0.12	0.92	0.09	0.74	1.18	—	—	—

Metal brackets	C-GM	1.0	0.07	0.96	0.13	0.86	1.07	2	16.44	<0.001
	GM	1.1	0.12	1.09	0.13	0.97	1.36	—	—	—
	SS	1.2	0.1	1.22	0.06	1.03	1.42	—	—	—

*⁣* ^*∗*^ANOVA and followed by post-hoc pairwise *t*-test.

**Table 4:** Pairwise *t*-test results for friction values of ceramic and metal brackets on coated GM, GM, and SS.

Bracket type	Group 1	Group 2	*p*-Value*⁣*^*∗*^
Ceramic brackets	GM	C-GM	1
	SS	C-GM	0.114
	SS	GM	0.671

Metal brackets	GM	C-GM	0.017
	SS	C-GM	<0.001
	SS	GM	0.033

*⁣* ^*∗*^Pairwise *t*-test with Bonferroni correction for multiple testing; *p*-value was set at 0.05.

**Table 5:** Summary of confocal profilometry.

Wire type	Root mean square (SQ) (µm)
C-GM	1.204
GM	0.509
SS	0.254

**Table 6:** Optical density (OD540) readings of bacterial counts.

Wire type	Mean	SD	Median	IQR	Min	Max	*p*-Value*⁣*^*∗*^		
							C-GM vs. GM	C-GM vs. SS	GM vs. SS
C-GM	0.61	0.11	0.60	0.15	0.44	0.88	<0.001	<0.001	<0.001
GM	1.07	0.08	1.07	0.12	0.95	1.28	—	—	—
SS	0.88	0.07	0.89	0.11	0.69	1.00	—	—	—

*⁣* ^*∗*^Pairwise *t*-test comparisons with Bonferroni correction for multiple testing; *p*-value set at 0.05.

## Data Availability

The data that support the findings of this study are available upon request from the corresponding author. The data are not publicly available due to privacy or ethical restrictions.
